# Submit to Pearls From the Pros

**DOI:** 10.1016/j.gastha.2025.100813

**Published:** 2025-09-22

**Authors:** David A. Katzka, Vinod K. Rustgi, Shanthi Srinivasan

**Affiliations:** Department of Medicine Division of Digestive and Liver Diseases, Columbia University Medical Center, Presbyterian Hospital, New York, New York; Rutgers Robert Wood Johnson Medical School, New Brunswick, New Jersey; Division of Digestive Diseases, Department of Medicine, Emory University School of Medicine, Atlanta, Georgia

Dear Readers,

We are inviting **ALL** clinicians to submit a “Pearls from the Pros” article to *Gastro Hep Advances*. Although the use of evidence-based medicine is ideal in the practice of medicine, important research in clinical medicine that requires evidence begins with an idea or observation often coming from the patient’s bedside. Yet, these pivotal physician observations may not be further explored nor published and only circulated to trainees or patients of the seasoned physician. The goal of this column is to allow the best of our clinical gastroenterologists and hepatologists to offer knowledge obtained after years of practice which they feel to be true but not further evaluated at this time through formal research methods. We recognize that there are seasoned gastroenterologists and hepatologists all over the world in every type of medical practice who have likely made important clinical observations that deserve mention in our journal and will improve patient care. We kindly ask you to follow the publication format outlined below, and your submission will be carefully reviewed by the Editors for possible publication. Thank you so much for participating, and we look forward to reading your great “pearls.”

• Articles should be no more than 200 words and may include relevant images.
• Articles will not be peer-reviewed but evaluated by journal editors at https://www.editorialmanager.com/ghadvances/
• Images and videos are encouraged!

For more information and submission requirements, please visit our Guide for Authors page.

Sincerely,

David Katzka, M.D.

Associate Editor, *Gastro Hep Advances*.

Vinod K. Rustgi, M.D.

Shanthi Srinivasan, M.D.

Editors-in-Chief, *Gastro Hep Advances*.

